# Indirect Prevention and Treatment of Depression: An Emerging Paradigm?

**DOI:** 10.32872/cpe.6847

**Published:** 2021-12-23

**Authors:** Pim Cuijpers

**Affiliations:** 1 Department of Clinical, Neuro and Developmental Psychology, Amsterdam Public Health research institute, Vrije Universiteit Amsterdam, Amsterdam, The Netherlands; Philipps-University of Marburg, Marburg, Germany

**Keywords:** depression, disease burden, indirect treatment, stigma, prevention

## Abstract

**Background:**

Although depression is one of the main public health challenges of our time, the uptake of interventions aimed at the prevention and treatment is low to modest. New approaches are needed to reduce the disease burden of depression.

**Method:**

Indirect prevention and treatment may be one method to increase uptake of services. Indirect interventions aim at problems related to depression but with lower stigma and prevent or treat depression indirectly. This paper describes the approach, the empirical support and limitations.

**Results:**

A growing number of studies focus on indirect prevention and treatment. Several studies have examining the possibilities to prevent and treat depression through interventions aimed at insomnia. Several other studies focus on indirect interventions aimed at for example stress and perfectionism. Digital ‘suites’ of interventions may focus on daily problems of for example students or the workplace and offer a broad range of indirect interventions in specific settings.

**Conclusion:**

Indirect prevention and treatment may be a new approach to increase uptake and reduce the disease burden of depression.

## Highlights

- Effective treatments and preventive interventions are available for depression but uptake is low.
- Indirect prevention and treatment focus on problems related to depression.
- Uptake of indirect interventions is higher because they focus on daily problems.
- Indirect interventions may provide a new paradigm for prevention and treatment.

Depressive disorders are highly prevalent (Alonso et al., 2004; Kessler & Bromet, 2013), have a high incidence (Waraich et al., 2004), and are associated with a substantial loss of quality of life for patients and their relatives (Saarni et al., 2007; Vos et al., 2016), increased mortality rates (Cuijpers et al., 2014), high levels of service use, and enormous economic costs (Greenberg et al., 2003; Smit et al., 2006). Major depression is currently ranked fourth worldwide in disease burden, and it is expected to rank first in high-income countries by the year 2030 (Mathers & Loncar, 2006). There is no doubt that depression is one of the most important public health challenges in the coming decades (Cuijpers et al., 2012).

One of the main problems that limits the impact of attempts to reduce the burden of disease of depression is the low uptake of treatments. This uptake is low in the general population, with rates that are often lower than 30%, even in high income countries (Chisholm et al., 2016). In some specific groups, such as adolescents, young adults, older adults and minority groups, the number of those seeking help is even considerably lower. For example, one study found that the uptake in college students in high income countries was only 30% for those with a 12-month depressive disorder (Bruffaerts et al., 2019). The low uptake of treatment is related to several factors, such as lack of financial resources and availability of clinicians. This is an important reason why the uptake of services is low in low– and middle-income countries, where hardly any infrastructure for mental health care exists, too few trained clinicians are available and insufficient resources are available to pay for these services. However, also in high-income settings the uptake is low because of the stigma related to depression, being unaware that existing problems are indeed depression, lack of time and the preference of many patients to manage their problems on their own or with friends and family.

The uptake of preventive services is even lower. In one study we estimated that about 1% of those meeting criteria for participation in indicated prevention actually participated, even when offered free or almost free of charge (Cuijpers et al., 2010). Reasons are comparable to those mentioned earlier for the low uptake of treatments and include stigma, and preference to solve problems her/himself instead of seeking help (Cuijpers et al., 2010).

Another limiting factor in the reduction of the disease burden of depression is that interventions aimed at the prevention and treatment of depression are effective, but their effects are modest. The impact of interventions can be seen as the product of the uptake and the effects, and when both the uptake and the effects are small, the impact is also small. Meta-analyses of psychological and pharmacological treatments usually find that the interventions improve outcomes in about 20 to 25% of patients, compared to control conditions (Cipriani et al., 2018; Cuijpers et al., 2021b). That means that most patients either improve regardless of treatment or do not respond to them (Cuijpers, 2018). Preventive interventions are also effective and can reduce the risk of developing a depressive disorder in the coming year with about 20% (Cuijpers et al., 2021a), but despite these positive effects the majority of high-risk participants still develop a disorder. Together with the low uptake of these services, it should not come as a surprise that the prevalence of depressive disorders has not been reduced over the past decades, despite the availability of services for prevention and treatment (Ferrari et al., 2013).

## The Indirect Approach to Prevention and Treatment

Conventional methods to increase help-seeking rates include universal mental health awareness campaigns (Salerno, 2016; Yamaguchi et al., 2013), gatekeeper training (Lipson et al., 2014) and specific interventions aimed at improving help-seeking behaviours (Ebert et al., 2019). An alternative method to increase uptake is what could be called “indirect” prevention and treatment. The basic idea of these “indirect” interventions is that they focus on problems related to depression, but not directly on depression itself. At the same time the participants learn techniques which not only directly affect the problem, but also have an effect on depression or may prevent future depressive symptoms or disorders. For example, people with insomnia and depression receive an intervention aimed at insomnia, but also learn skills to manage their mood. Insomnia is less stigmatising than depression to talk about or to seek treatment for, and if the intervention aimed at insomnia is also effective in reducing depression, then the participant is still successfully “treated” for depression in an indirect way.

The same idea can be applied to prevention. If someone with insomnia has subthreshold depression but no diagnosis for a depressive disorder, this person meets criteria for participation in an indicated prevention program. An intervention aimed at insomnia for this person could be considered as indicated prevention and has the potential to prevent the onset of depressive disorders in an indirect way. Again, participation in an intervention on insomnia is probably less stigmatising as an intervention to prevent a depressive disorder.

## Research on the Indirect Approach

A growing number of studies is focusing on this strategy. For example, recent studies have shown that cognitive behaviour therapy for insomnia in patients with both insomnia and depression, reduces not only insomnia but also depression (van der Zweerde et al., 2019). The effect sizes found for such interventions on depressive symptomatology are comparable to those of ‘regular’ treatment of depression. This is true even though the interventions are not directly aimed at depression, and the stigma to participate in interventions for insomnia is lower than interventions for depression. The generic cognitive behavioural strategies that participants learn for handling insomnia, are in many ways comparable to those that are used in cognitive behavioural therapies for depression. Or it could be the case that improvement of insomnia is the first step to escape from a vicious circle of mood problems. Other research has used the same principle as a preventive strategy. For example, one study found that participants with insomnia and subthreshold depression who receive cognitive behaviour therapy for insomnia had a smaller chance to develop major depression at follow-up (Christensen et al., 2016).

But this principle of ‘indirect’ prevention and treatment of depression is not limited to insomnia. One recent study examined the effects of an intervention aimed at perinatal women scoring high on perfectionism, with depression and anxiety as an outcome (Lowndes et al., 2019). This study found that the intervention significantly reduced perfectionism, and path analyses demonstrated a significant indirect effect of the intervention on depression and anxiety. Another study showed that a considerable part of the participants in interventions aimed at ‘stress management’ also suffer from depression, and that the effects of this stress management training on depression were considerable and comparable to the effects of psychological treatments of depression in general (Weisel et al., 2018). Interventions aimed at problems like perfectionism, procrastination, and low self-esteem have also been found to have considerable effects on depression in those suffering from depression at baseline (Cuijpers et al., 2021). There is much research on interventions for such common psychological problems and for many high-risk groups, but not with a focus on indirect prevention and treatment of depression.

One could argue that this approach is very similar to selective prevention. Selective prevention is aimed at people who have an increased risk to develop a depressive disorder. Selective interventions are for example aimed at children of depressed parents (Clarke et al., 2001), pregnant women with an increased risk for postpartum depression (Phipps et al., 2013; Zlotnick et al., 2016), dementia caregivers (Cheng et al., 2020) or patients with general medical disorders (Rovner et al., 2014). Interventions aimed at these high-risk groups may support participants in coping with their problems but may at the same time prevent or reduce existing depressive symptomatology. However, these studies usually first have the intention to support participants with their problems and have depression only as secondary outcome. They are hardly ever designed as indirect treatment or prevention of depression in the sense that they report the number of depressed participants at baseline and the effects of the intervention on depression in these participants.

## “Suits” of Indirect Interventions for Specific Settings

One important development in recent years may help in disseminating indirect interventions. Internet-based cognitive behavioural interventions have been developed for many different disorders, problems and target groups. Because they can be easily adapted and broadly disseminated it could be possible to develop ‘suites’ of multiple interventions for problems with relatively low stigma that are related to depression. For example, it could be possible to develop a suite of interventions for college students on procrastination, perfectionism, low self-esteem, test-anxiety, stress, worry, and any other common problem that is brought forward by students themselves. Or a suite of interventions for employees in large companies on stress, conflict resolution, assertiveness, time management and problem-solving. Comparable suites could be developed for high-school students, perinatal women, or specific groups of patients in general hospitals. Because such interventions are scalable and not expensive after first development, they could be offered to full populations, but are in fact meant to be early interventions for depression and potentially other common mental disorders.

## Challenges and Limitations

It is possible that indirect interventions can be an option for mild depression, but not for moderate and severe depression. However, not every person with severe depression gets treatment, and it is very well possible that they are willing to participate in these ‘indirect’ interventions. It is an empirical question whether indirect treatments in these patients are still better than the current practice of not providing treatment at all if the patient cannot be motivated to get treatment. How other clinical issues, such as suicidality and comorbidity, should be handled is also not yet clear. If patients participate in interventions that are not directly aimed at depression, who will take care of suicide risks and correct diagnoses? In order to avoid risks in these domains extended baseline assessment could be needed for these indirect interventions. It is also uncertain whether such an approach would indeed lead to higher uptake rates of services. Will interventions aimed at reducing insomnia, perfectionism or stress lead indeed to better outcomes then just offering mental health services? These are empirical question that have to be answered with future research, but at ‘face value’ they can lead to a higher uptake, especially when they are offered as ‘suits’ of interventions.

## Conclusion

Depression is a highly heterogeneous condition with largely varying symptoms patterns and associations with other variables. This heterogeneity is typically seen as problematic and hampering progress in our understanding and management of depression. But it may also offer new possibilities for indirect prevention and treatment. A growing number of studies focuses on problems related to depression, and interventions focus not directly on depression itself, but participants learn techniques that not only affect such problems directly, but also depression. This may offer new possibilities to get effective interventions to people who usually do not get treatment for depression. Much research is needed to examine whether this is possible, feasible and effective, but the first findings are hopeful. Maybe we are witnessing the start of a new paradigm in the prevention and treatment of depression.
